# IL-17A induces heterogeneous macrophages, and it does not alter the effects of lipopolysaccharides on macrophage activation in the skin of mice

**DOI:** 10.1038/s41598-017-12756-y

**Published:** 2017-09-29

**Authors:** Kozo Nakai, Yu-Ying He, Fumiko Nishiyama, Fukiko Naruse, Reiji Haba, Yoshio Kushida, Naomi Katsuki, Tetsuya Moriue, Kozo Yoneda, Yasuo Kubota

**Affiliations:** 1 Department of Dermatology, Kagawa University, Kagawa, Japan; 2 Department of Medicine, Section of Dermatology, University of Chicago, Chicago, USA; 3 Department of Diagnostic Pathology, Kagawa University, Kagawa, Japan; 4 Department of Pharmacology, Osaka Ohtani University, Osaka, Japan

## Abstract

Macrophages are central to inflammatory response and become polarized towards the M1 or M2 states upon activation by immunostimulants. In this study, we investigated the effects of lipopolysaccharides (LPS) and interleukin (IL)-17A on the activation of macrophages in *in vivo* mouse skin. We examined whether macrophages are activated in the skin of imiquimod (IMQ)-treated mice, a model for IL-17A-induced psoriasis-like skin inflammation, and flaky-tail (*Flg* ^*ft*^) mice, a model for IL-17A-induced chronic atopic dermatitis-like skin inflammation. LPS and IL-17A independently increased the expression levels of iNOS, CX3CR1, CD206, phospho-STAT1 and phospho-STAT3 proteins in the skin of B6 mice, and the effects of LPS was not altered by IL-17A. The expression levels of these proteins were increased in the skin of IMQ-treated and *Flg* ^*ft*^ mice. IL-17A neutralization increased the expressions of iNOS and phospho-STAT1 in the IMQ-treated skin, but it decreased the expressions of CD206 and phospho-STAT3 proteins in the skin of *Flg* ^*ft*^ mice, suggesting that macrophages to change from the M2 to the M1 state in the skin of these mice. These results suggest that IL-17A is involved in the activation of macrophages that are in the process of adopting the heterogeneous profiles of both the M1 and M2 states.

## Introduction

In skin, various immune cells are activated by invading pathogens or skin damage. Macrophages are vital to innate immunity, and their activation leads to an increased capacity to regulate other cells through the release of cytokines and chemokines that is induced in adaptive immunity^1^. Persistent macrophage activity results in the development of chronic inflammatory skin diseases such as psoriasis and atopic dermatitis (AD)^2^. Abnormal immune conditions of tissues affects the activation and plasticity/polarization of macrophages (i.e. their maturation towards the M1 or M2 state)^3^. The classical pathway is defined as the interferon (IFN)-γ-dependent activation of M1 type macrophages by T helper 1 (Th1)-type responses. The alternative pathway is defined as the M2 type macrophage activation by Th2-type cytokines interleukin (IL)-4, IL-6, IL-10 and IL-13. However, recent studies have shown that the macrophage lineages are diverse and heterogeneous as demonstrated by the presence of unique M1/M2 heterogeneous macrophages in IL-17 immunity.

IL-17 family members play an active role in inflammatory diseases and autoimmune diseases^4^. IL-17A is the prototype pro-inflammatory cytokine of the IL-17 family^5^, which is largely produced by activated memory T lymphocytes, and functions to stimulate innate immunity. A well-known IL-17A-related skin disease is psoriasis. The association between IL-17A and psoriasis is well established in humans, mouse and *in vitro* models^6^. The roles of IL-17A are also implicated in the pathogenesis of AD^7,8^. IL-17A is highly expressed in flaky-tail (*Flg* ^*ft*^) mouse, a murine AD model, that carries mutations in the *filaggrin* gene^9,10^. Moreover, IL-17A is involved in infectious, inflammatory, neutrophilic, granulomatous, bullous and malignant skin diseases^11^. However, there are few reports regarding the direct effects of IL-17A on the macrophage function in the skin.

To clarify whether macrophages act as effectors of Th17 immunity, we investigated the effects of IL-17A injection on the macrophage activation in the skin of B6 wild-type mice. Lipopolysaccharides (LPS) is known to activate macrophages via Toll-like receptor 4 (TLR4) related classical pathway. IL-17A in addition to LPS enhances proinflammatory cytokine expression in human carotid plaques, suggesting the synergistic effects of IL-17A and LPS on macrophage activation^12^. However, tumor necrosis factor (TNF)-α, which could be elevated in IL-17A-induced inflammation condition, induces a tolerant state in macrophages with less cytokine production by LPS^13^. These reports lead us to investigate an additive effect of LPS on the IL-17A-mediated inflammatory condition in *in vivo* skin.

Next, we examined whether macrophages are activated in the skin of a murine psoriasis model (imiquimod (IMQ)-treated mouse) and a murine AD model (*Flg* ^*ft*^ mouse), and we examined the effects of LPS on macrophage activations in these model mice. We also treated these model mice with IL-17A neutralization.

## Materials and Methods

### Antibodies

The anti-F4/80 antibody was purchased from Bio-Rad (Hercules, CA, USA). The anti-iNOS antibody was purchased from BD Transduction Laboratories (San Jose, CA, USA), Thermo Scientific (Fremont, CA, USA) or Abcam (Cambridge Science Park, Cambridge, UK). CX3CR1 was obtained from Bioss (Woburn, Massachusetts, USA). CD68 was obtained from Abcam and CD206 from Bioss, BIO-RAD (Hercules, CA, California) or R&D Systems (Minneapolis, MN, USA). Antibodies against phospho-STAT1, STAT1, phospho-STAT3, STAT3 and β-actin were purchased from Cell Signaling Technology (Danvers, MA, USA).

### Mice and Treatments

All animal experiments and methods were approved by the Kagawa University Institutional Animal Care and Use Committee. Mice were handled in compliance with the guidelines for conducting Animal Experiments at Kagawa University. The *Flg* ^*ft*^ mice were obtained from the Jackson Laboratory (Bar Harbor, ME USA), and the C57BL/6 J (B6) mice were obtained from CLEA Japan (Megro, Tokyo, Japan). The *Flg* ^*ft*^ mice were backcrossed three generations into a B6 strain background. The mice were housed in a SPF facility at the Health Science Center of Kagawa University under controlled temperature (23 °C) and humidity (55%). The mice were given food and water *ad libitum* (MF: Oriental Yeast Co., Itabashi, Tokyo, Japan). Mouse IL-17A (ProSpec, East Brunswick, NJ, USA) was administered intraperitoneally (0.25 μg) once a day for a week or subcutaneously (3 μg) in the skin of B6 mice once 24 hours before LPS injection. A daily topical dose of 60 mg of commercially available IMQ cream (5%) (Beselna Cream^®^; Mochida Pharmaceuticals, Tokyo, Japan) was applied to the shaved back skin once a day for a week. Mouse anti-mouse IL-17A antibody (clone: 17F3; Bio X Cell, West Lebanon, NH, USA) was administered intraperitoneally (50 μg) once a day for a week. LPS (Sigma, St. Louis, MO, USA, 0.1 mg) was injected subcutaneously into the dorsal area of the mice. Six hours later, the mice were sacrificed by intraperitoneal sodium pentobarbital (1 g/kg) injection, and the full thickness sections of the dorsal skin were subsequently dissected. Mice were sacrificed within 5–12 weeks in age. Groups of four to six mice were used in each experiment. Each experiment was repeated a minimum of two times.

### Immunohistochemical analysis

Skin samples (1–2 cm^2^) that were removed from the mid-dorsum of mice were directly fixed in 10% buffered formalin. Tissues were dehydrated and embedded in paraffin prior to cutting 2–5-μm thick sections. The samples were subsequently deparaffinized in xylene, rehydrated and immunostained with primary antibodies (1:200 dilution) using the Histofine simple stain reagent (Nichirei, Tokyo, Japan) according to the manufacturer’s protocol. Briefly, endogenous peroxidase activity was blocked by incubating the sections with 3% hydrogen peroxide for 5 min. After a wash with PBS, the sections were incubated with the primary antibodies at room temperature for 1 h, washed in PBS and subsequently incubated with peroxidase-conjugated secondary antibodies. After wash, sections were incubated in a 3–3′-diaminobenzidine tetrahydrochloride solution and counterstained with Mayer’s hematoxylin. The number of positively stained cells was counted in 4 different high-power fields (HPF; ×400).

### Human atopic dermatitis and psoriasis samples

All human specimens and all experimental protocols were studied after approval by the Kagawa University and University of Chicago Institutional Review Board. Informed consent was obtained from all subjects. All methods were carried out in accordance with relevant guidelines and regulations. Formalin-fixed paraffin-embedded tissue blocks were obtained from the tissue bank archives at the Kagawa University. AD and psoriasis specimens were used for immunohistochemical analysis of CD68, CD206 and iNOS protein expression. TripleStain IHC kit (Abcam) was utilized to examine the colocalization of CD206 and iNOS protein.

### Immunofluorescence microscopy on frozen tissue sections

Frozen tissue sections (30 µm) were cut and placed onto Superfrost plus slides. Sections were pre-blocked in a solution containing 1% BSA, 0.1% Triton X-100, and 1% gelatin in PBS. Primary antibodies (1:200 dilution) were then added to a fresh solution and incubated with the samples at room temperature for 1 h. After washing the slides 3 times with PBS for 10 min each, the sections were incubated with fresh solution containing secondary FITC-conjugated antibodies (1:400 dilution) or Alexa Fluor 555-conjugated antibodies (1:400 dilution) for 30 min before washing and mounting. Nuclei were stained with TO-PRO-3. Sections were examined using a confocal microscope (LSM 700; Carl Zeiss, Jena, Thüringen, Germany).

### Western blot analysis

Skin tissue specimens were homogenized with a Polytron homogenizer in RIPA buffer containing 1% v/v NP40, 20 mM Tris (pH 7.7), 150 mM NaCl, 1 mM EDTA and a mixture of protease inhibitors (Calbiochem, San Diego, CA, USA). After incubation at 4 °C for 20 min, the samples were sonicated on ice and centrifuged at 14,000 rpm for 10 min. The protein concentration of the supernatants was analyzed using the BCA protein assay (Pierce, Rockford, IL, USA). An equal amount of protein was separated on reducing NuPAGE 4–12% Bis-Tris or 7% Tris-acetate gel (Invitrogen, Carlsbad, CA, USA) and transferred to a nitrocellulose membrane. Alternatively, the protein was separated on SDS-PAGE gel and transferred to a PVDF membrane. The membrane was then probed with primary antibodies. Membrane-bound primary antibodies were visualized using the appropriate secondary antibodies conjugated to horseradish peroxidase and a chemiluminescent substrate (Pierce, Rockford, IL, USA). The immunoreactive signals from the chemiluminescent substrate were visualized by exposure to standard X-ray films. Images were subjected to densitometric analysis using the Image J software (National Institutes of Health, Bethesda, MD, USA).

### Quantitative RT-PCR

Total RNA was extracted from the skin tissues using TRIzol reagent (Invitrogen, Carlsbad, CA, USA). Reverse transcription was performed using a high-capacity cDNA reverse transcription kit (Applied Biosystems, Foster City, CA, USA). Quantitative RT-PCR was performed using the Taqman gene expression assay system (Applied Biosystems, Foster City, CA, USA). The following probes were used: Mm0045259_m1 (IL-4), Mm00446190_m1 (IL-6), Mm01288386_m1 (IL-10), Mm00434204_m1 (IL-13), Mm00439618_m1 (IL-17a), Mm01160011_g1 (IL-23), Mm00439552_s1 (IFN-β), Mm01168134_m1 (IFN-γ), Mm00443258_m1 (TNF-α), Mm00436450_m1 (CXCL2), and Mm99999915_g1 (GAPDH). Probes for GAPDH were used as an endogenous control. Quantitative RT-PCR was carried out using an ABI 7500 real-time PCR system. Gene expression values were calculated based on the comparative threshold cycle method, normalized to GAPDH expression, and displayed as a fold induction relative to the control.

### Statistical analysis

Data are expressed as the mean ± S.E. Statistical analysis was performed by an analysis of variance followed by Mann-Whitney’s U-test. P-values of <0.05 were considered significant.

## Results

### Treatment with subcutaneous IL-17A injection activated macrophages in the skin of B6 mice

**Figure 1:**
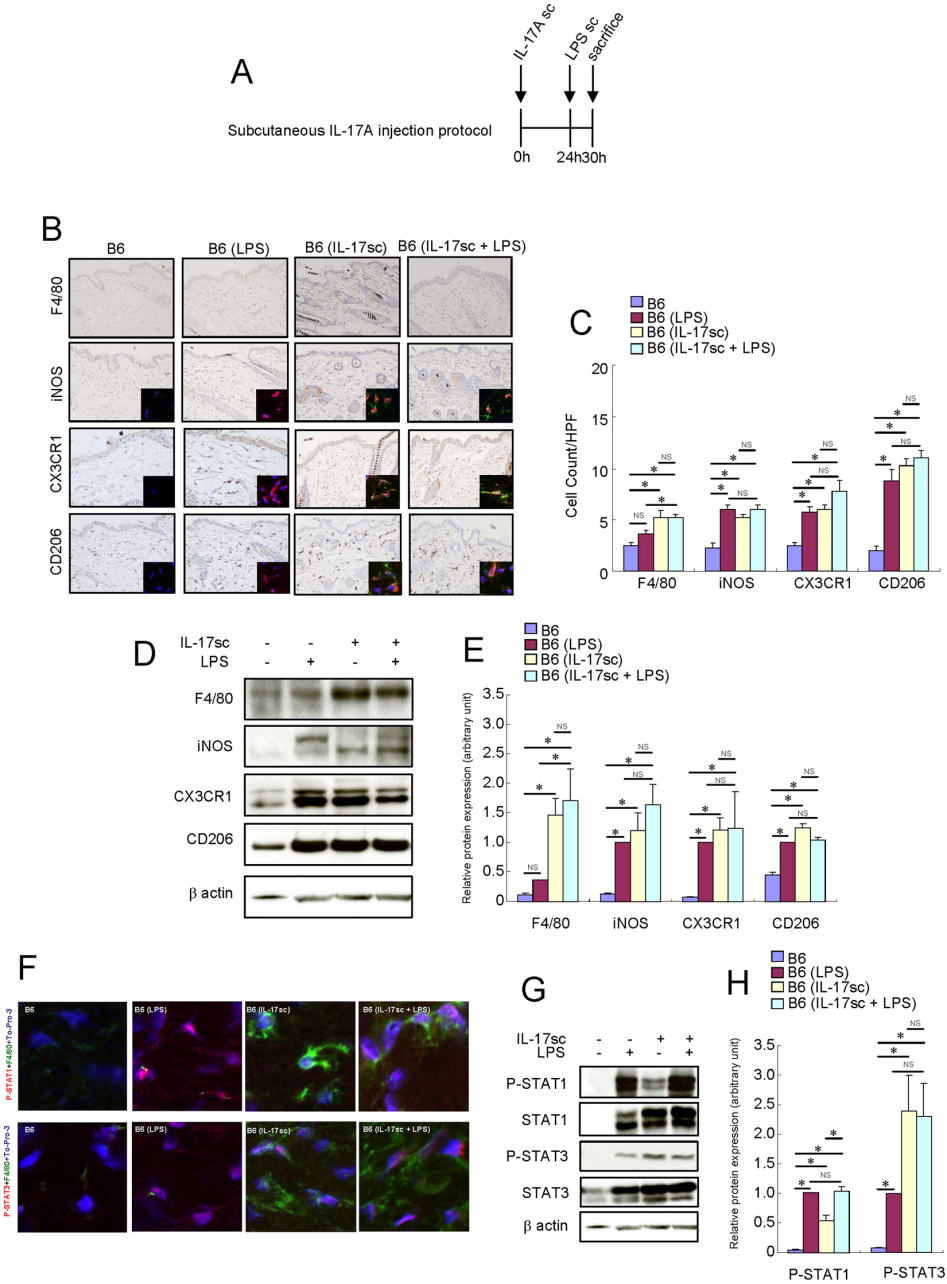
Short-term treatment with local IL-17A activated resident macrophages in the skin of B6 mice. Mouse recombinant IL-17A was administered subcutaneously (3 μg) once a day in the skin of B6 mice. LPS (0.1 mg) was injected subcutaneously into the dorsal area of the mice. Six hours later, the mice were sacrificed, and the full thickness sections of the dorsal skin were dissected. (**A**) Schema of subcutaneous IL-17A injection protocol. (**B**) Immunohistochemistry of F4/80, iNOS, CX3CR1 and CD206 protein expression in the skin of B6 mice. Immunofluorescence co-staining images for iNOS (red), CX3CR1 (red) and CD206 (red) with F4/80 (green) and To-Pro-3 (blue) are shown. Due to the low expression of F4/80 protein in the skin of B6 and LPS-injected B6 mice, the F4/80 immunofluorescence signal of these mice is barely observed in the condition of this figure. (**C**) Number of cells/HPF (×400). (**D**) Representative Western blot results of F4/80, iNOS, CX3CR1, CD206 and β-actin are shown. (**E**) Densitometric analysis results were obtained from pooled data. Relative protein expression normalized to β-actin, arbitrary units. (**F**) Immunofluorescence co-staining images of phospho-STAT1 (red) and phospho-STAT3 (red) with F4/80 (green) and To-Pro-3 (blue). Due to the low expression of F4/80 protein in the skin of B6 and LPS-injected B6 mice, the F4/80 immunofluorescence signal of these mice is barely observed in the condition of this figure. (**G**) Representative Western blot results of phospho-STAT1, phospho-STAT3 and β-actin are shown. (**H**) Densitometric analysis results were obtained from pooled data. Relative protein expression normalized to β-actin, arbitrary units. Values represent the mean ± S.E. (n = 4-6). *P < 0.05. NS: not significant.

To test the hypothesis that IL-17A activates resident macrophages, we injected IL-17A only or in combination with subsequent LPS subcutaneously (Fig. 1A). LPS significantly increased the number of iNOS^+^, CX3CR1^+^ and CD206^+^ cells in the skin of B6 mice (Fig. 1B,C). Furthermore, the protein expression levels of iNOS, CX3CR1 and CD206 increased after LPS injection (Fig. 1D,E). LPS also increased the expression levels of both STAT1 and STAT3 and their phosphorylation (Fig. 1F,G,H) in the mouse skin tissue. Subcutaneous local IL-17A injection significantly increased the number of F4/80^+^ cells, suggesting an increase in the number of matured resident macrophages (Fig. 1B,C). Furthermore, the number of iNOS^+^, CX3CR1^+^, and CD206^+^ cells increased in the skin of IL-17A-treated B6 mice (Fig. 1B,C). The protein expression levels of F4/80, iNOS, CX3CR1 and CD206 in the mouse skin tissue were also elevated after the subcutaneous injection of IL-17A (Fig. 1D,E). Both STAT1 and STAT3 are phosphorylated in the F4/80^+^ cells of the IL-17A-injected mouse skin (Fig. 1F). The expression levels of both STAT1 and STAT3 and their phosphorylation were increased in the IL-17A-injected mouse skin tissue (Fig. 1G,H).

Subcutaneous local IL-17A did not alter the effects of LPS on macrophage activation. These results suggest that subcutaneous local IL-17A and LPS non-redundantly activated skin macrophages into the M1/M2 heterogeneous phenotype.

### Treatment with systemic IL-17A-activated macrophages in the skin of B6 mice

**Figure 2:**
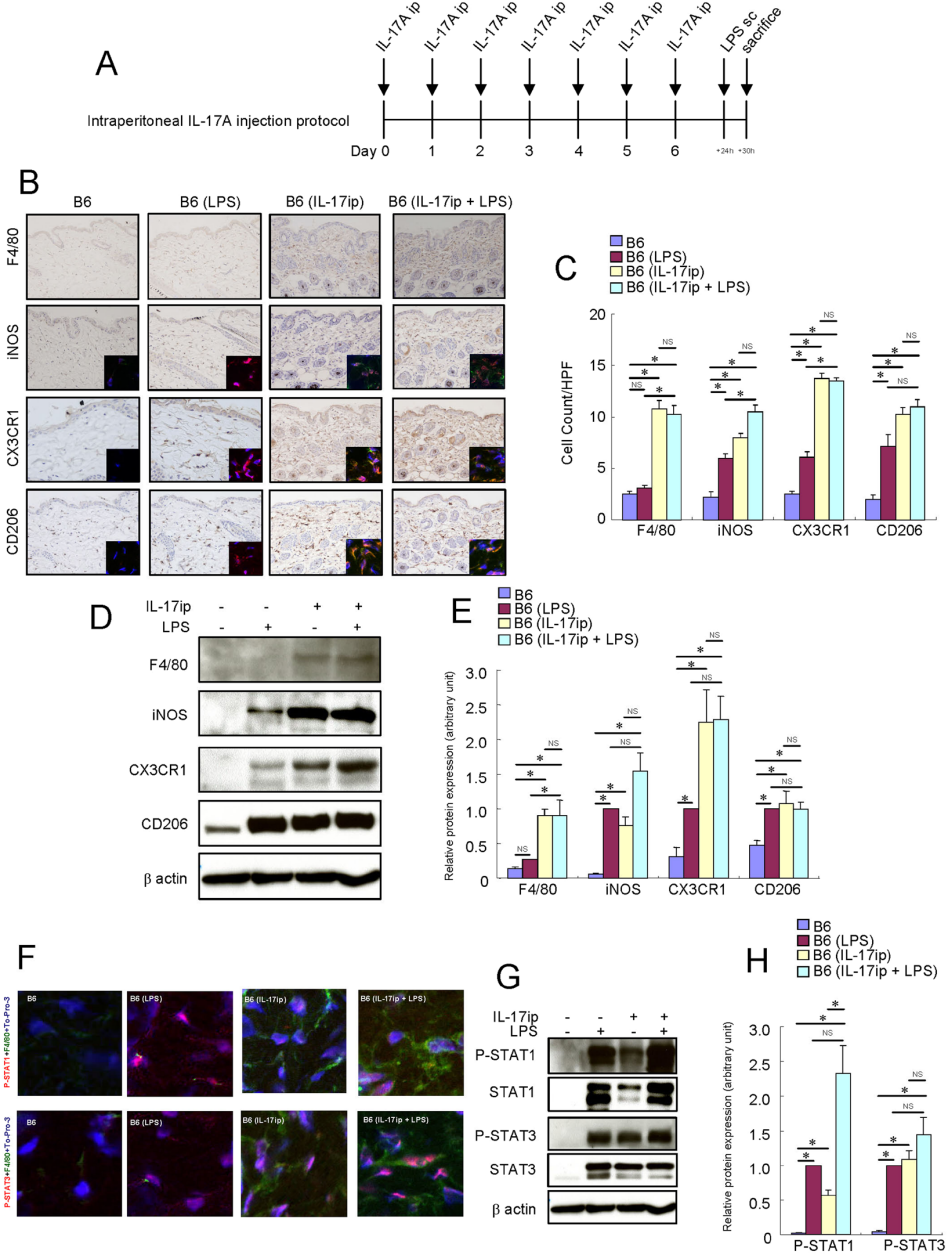
Long-term treatment with systemic IL-17A-activated macrophages in the skin of B6 mice. Mouse recombinant IL-17A was administered intraperitoneally (0.25 μg) once a day for a week in the skin of B6 mice. LPS (0.1 mg) was injected subcutaneously into the dorsal area of the mice. Six hours later, the mice were sacrificed, and the full thickness sections of the dorsal skin were dissected. (**A**) Schema of intraperitoneal IL-17A injection protocol. (**B**) Immunohistochemistry of F4/80, iNOS, CX3CR1 and CD206 protein expression in the skin of B6 mice. Immunofluorescence co-staining images of iNOS (red), CX3CR1 (red) and CD206 (red) with F4/80 (green) and To-Pro-3 (blue) are shown. (**C**) Number of cells/HPF (×400). Due to the low expression of F4/80 protein in the skin of B6 and LPS-injected B6 mice, the F4/80 immunofluorescence signal of these mice is barely observed in the condition of this figure. (**D**) Representative results of Western blotting of F4/80, iNOS, CX3CR1, CD206 and β-actin are shown. (**E**) Densitometric analysis results were obtained from pooled data. Relative protein expression normalized to β-actin, arbitrary units. (**F**) Immunofluorescence co-staining images of phospho-STAT1 (red) and phospho-STAT3 (red) with F4/80 (green) and To-Pro-3 (blue). Due to the low expression of F4/80 protein in the skin of B6 and LPS-injected B6 mice, the F4/80 immunofluorescence signal of these mice is barely observed in the condition of this figure. (**G**) Representative Western blot results of phospho-STAT1, phospho-STAT3 and β-actin are shown. (**H**) Densitometric analysis results were obtained from pooled data. Relative protein expression normalized to β-actin, arbitrary units. Values represent the mean ± S.E. (n = 4–6). *P < 0.05. NS: not significant.

To determine the effects of systemic IL-17A on macrophages in the skin, we treated the B6 mice with a daily intraperitoneal IL-17A injection for a week and examined whether the macrophages were activated in the skin (Fig. 2A). Intraperitoneal systemic IL-17A injection significantly increased the number of F4/80^+^, iNOS^+^, CX3CR1^+^ and CD206^+^ cells (Fig. 2B,C) and their respective protein expression in the mouse skin tissue (Fig. 2D,E). Both STAT1 and STAT3 were phosphorylated in the F4/80^+^ cells of the mouse skin by intraperitoneal IL-17A injection (Fig. 2F). Systemic IL-17A increased the expression levels of both STAT1 and STAT3 and their phosphorylation in the mouse skin tissue (Fig. 2G,H). Systemic IL-17A did not alter the effects of LPS on macrophage activation. These results suggest that long-term treatment with systemic IL-17A also activates macrophages in the skin of B6 mice in a different way from the LPS-induced macrophage activation.

### Macrophages were activated in the IMQ-treated skin of B6 mice

**Figure 3:**
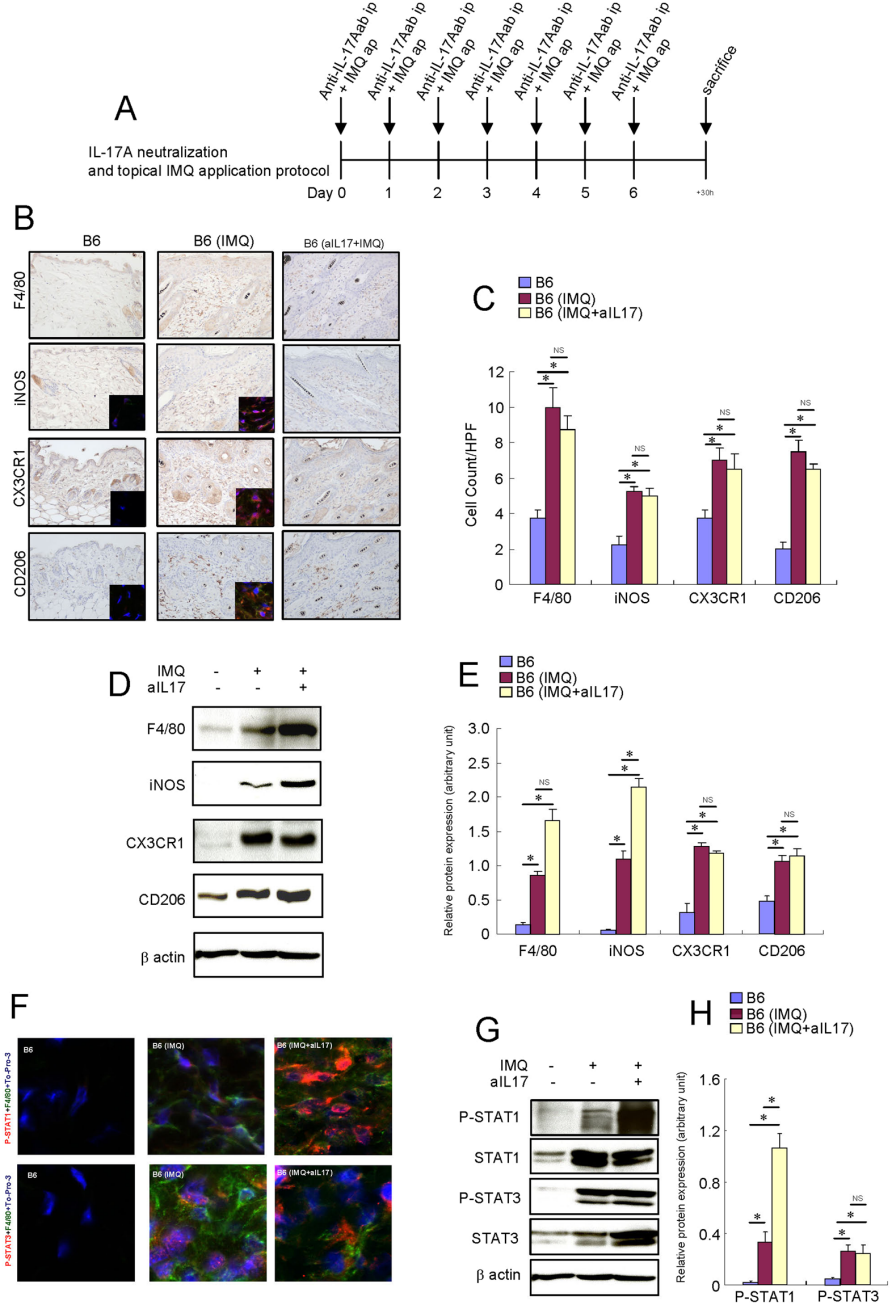
Macrophages were activated in the IMQ-treated skin of B6 mice. Mice were topically treated with a 60 mg daily topical dose of IMQ cream (5%). Mouse recombinant anti-mouse IL-17A was administered intraperitoneally (50 μg) once a day for a week in the IMQ-treated B6 mice. (**A**) Schema of intraperitoneal IL-17A injection and topical IMQ application protocol. (**B**) Immunohistochemistry of F4/80, iNOS, CX3CR1 and CD206 protein expression in the skin of B6 mice and IMQ-treated mice. Immunofluorescence co-staining images of iNOS (red), CX3CR1 (red) and CD206 (red) with F4/80 (green) and To-Pro-3 (blue) are shown. (**C**) Number of cells/HPF (×400). Due to the low expression of F4/80 protein in the skin of B6 mice, the F4/80 immunofluorescence signal of these mice is barely observed in the condition of this figure. (**D**) Representative Western blot results of F4/80, iNOS, CX3CR1, CD206 and β-actin are shown. (**E**) Densitometric analysis results were obtained from pooled data. Relative protein expression normalized to β-actin, arbitrary units. (**F**) Immunofluorescence co-staining images of phospho-STAT1 (red) or phospho-STAT3 (red) with F4/80 (green) and To-Pro-3 (blue). Due to the low expression of F4/80 protein in the skin of B6 mice, the F4/80 immunofluorescence signal of these mice is barely observed in the condition of this figure. (**G**) Representative Western blot results of phospho-STAT1, phospho-STAT3 and β-actin are shown. (**H**) Densitometric analysis results were obtained from pooled data. Relative protein expression normalized to β-actin, arbitrary units. Values represent the mean ± S.E. (n = 4–6). *P < 0.05. NS: not significant.

To determine whether macrophages are activated in the skin of psoriasis, we treated mouse skin with topical IMQ to induce transient psoriatic skin inflammation that is heavily dependent on IL-17 immunity (Fig. 3A)^14^. The protein expression levels of F4/80, iNOS, CX3CR1 and CD206 increased, and the number of F4/80^+^, iNOS^+^, CX3CR1^+^ and CD206^+^ cells markedly increased in the IMQ-treated skin of B6 mice (Fig. 3B–E). Both STAT1 and STAT3 were phosphorylated in the F4/80^+^ cells of the IMQ-treated skin of B6 mice (Fig. 3F). Both STAT1 and STAT3 proteins were increased and phosphorylated in the IMQ-treated skin tissue of B6 mice (Fig. 3G,H). These results suggest that macrophages are spontaneously activated in the skin of murine psoriasis models.

### IL-17A neutralization increased the protein expression levels of iNOS in the IMQ-treated skin of B6 mice

**Figure 4:**
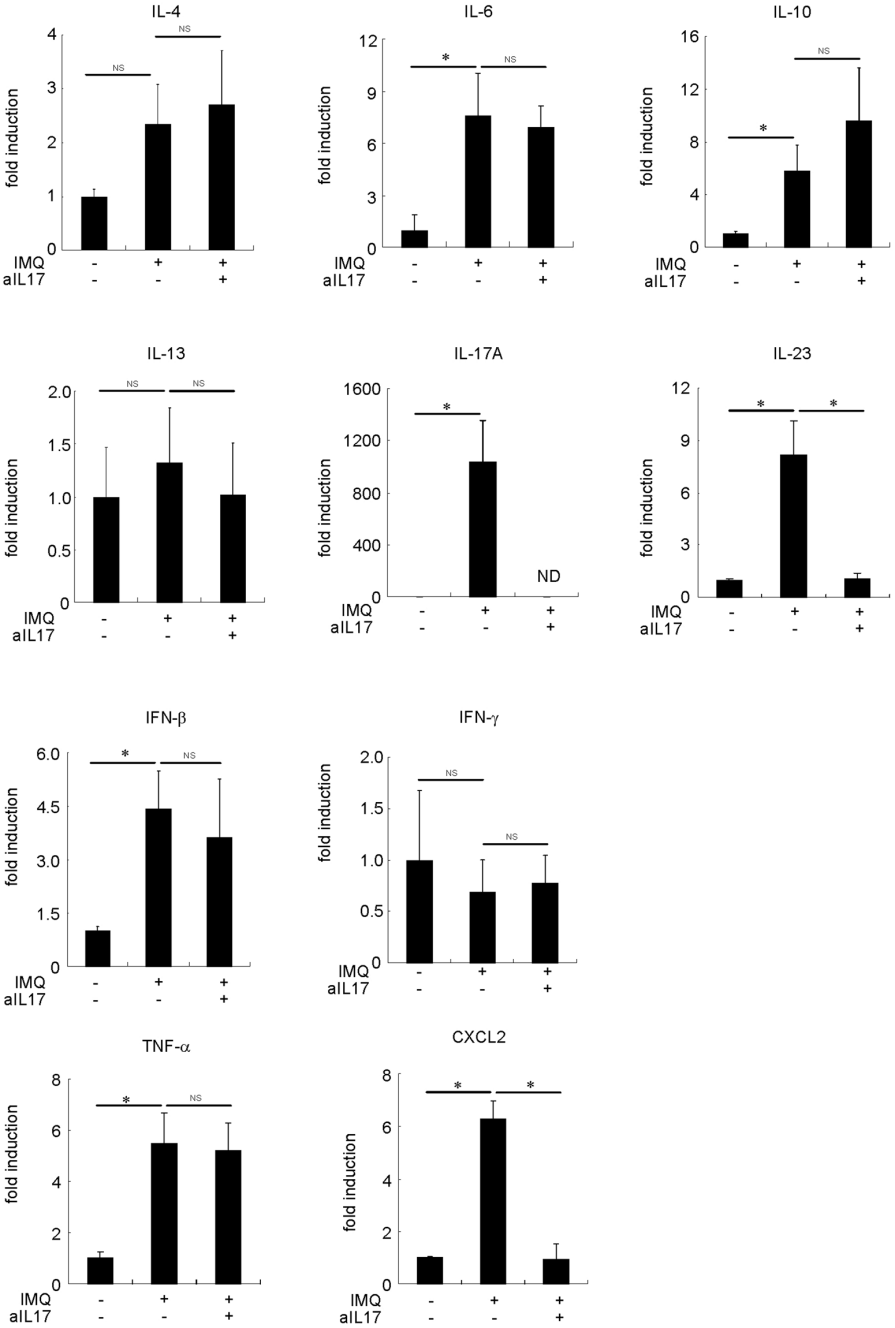
Cytokine, CXCL2 and TNF-α mRNA expression in the skin of IMQ-treated B6 mice and the effects of IL-17A neutralization. Mouse recombinant anti-mouse IL-17A was administered intraperitoneally (50 μg) and IMQ was topically applied once a day for a week B6 mice. The mice were sacrificed, and the full thickness sections of the dorsal skin were dissected. Quantitative RT-PCR analysis of cytokine, CXCL2 and TNF-α mRNA expression in the skin of IMQ-treated and -untreated B6 mice (n = 4–6). *P < 0.005. NS: not significant. ND: no data.

To determine whether the suppression of IL-17A can affect the phenotype of macrophages, we treated the B6 mice with IMQ and anti-IL-17A antibody for a week (Fig. 3A). Treatment with anti-IL-17A antibody did not alter the number of F4/80^+^, iNOS^+^, CX3CR1^+^ and CD206^+^ cells in the IMQ-treated skin of B6 mice (Fig. 3B,C). Although the protein expression levels of F4/80, CX3CR1 and CD206 were not altered, the protein expression levels of iNOS were increased in the mouse skin tissue (Fig. 3D,E). Furthermore, anti-IL-17A antibody treatment increased the activation of STAT1 in the IMQ-treated skin of B6 mice, but it did not alter the activation of STAT3 (Fig. 3G,H). We examined proinflammatory and proresolving mediators that may alter the macrophage phenotype in the IMQ-treated skin of B6 mice. The expression levels of IL-6, IL-17A, IL-23, IFN-β, TNF-α and CXCL2 were increased in the IMQ-treated skin of B6 mice (Fig. 4). After treatment with anti-IL-17A antibodies, the expression levels of IL-23 and CXCL2 were decreased in the IMQ-treated skin of B6 mice. These results suggest that the IMQ-induced modification of the macrophage phenotype is mostly independent of IL-17A itself in the skin of mice.

### Macrophages were activated in the skin of *Flg*^*ft*^ mice

**Figure 5:**
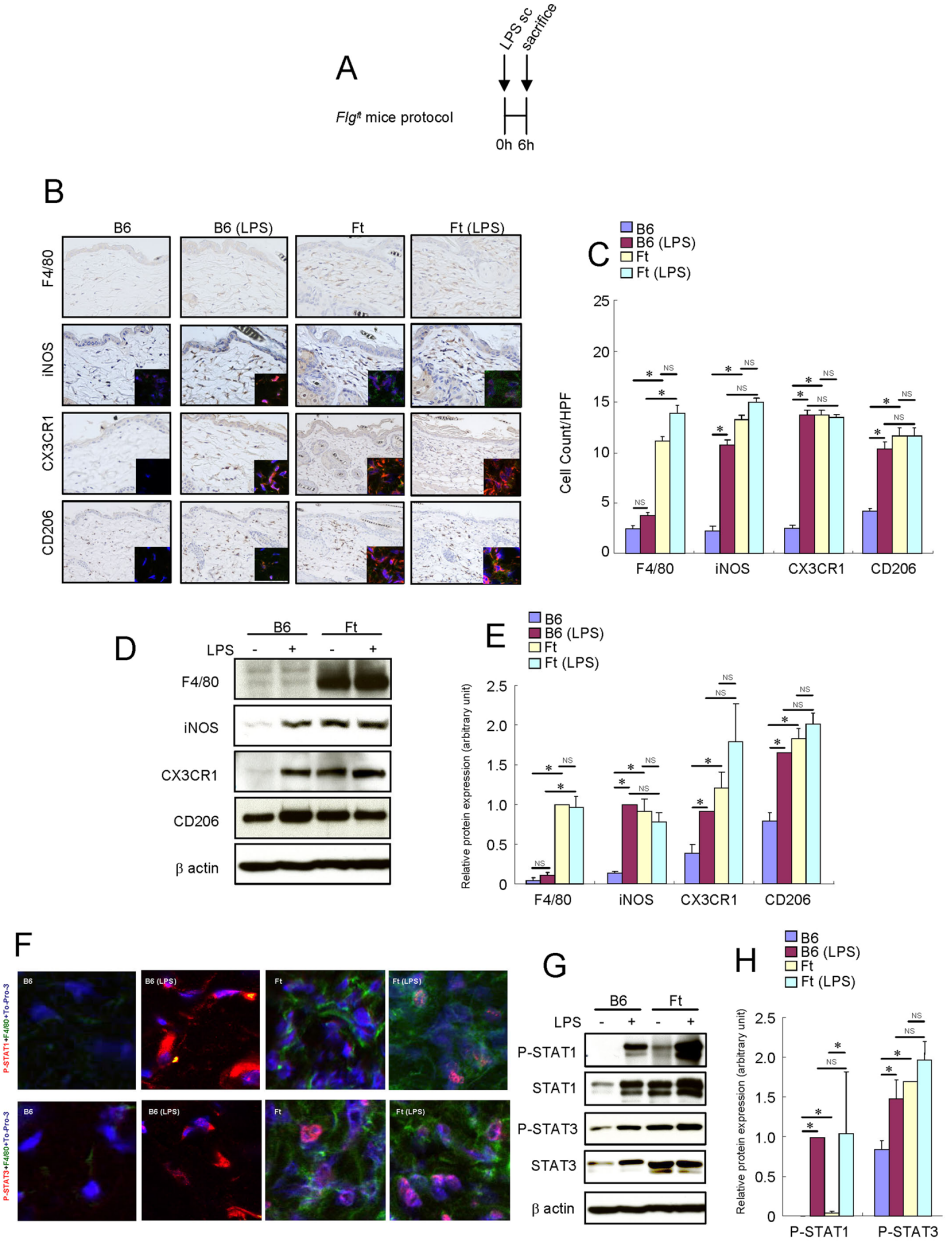
Macrophages were activated in the skin of *Flg* ^*ft*^ mice. LPS (0.1 mg) was injected subcutaneously into the dorsal area of the mice. Six hours later, the mice were sacrificed, and the full thickness sections of the dorsal skin were dissected. (**A**) Schema of protocol. (**B**) Immunohistochemistry of F4/80, iNOS, CX3CR1 and CD206 protein expression in the skin of B6 mice and *Flg* ^*ft*^ mice. Immunofluorescence images of iNOS (red), CX3CR1 (red) and CD206 (red) with F4/80 (green) and To-Pro-3 (blue) are shown. Due to the low expression of F4/80 protein in the skin of B6 and LPS-injected B6 mice, the F4/80 immunofluorescence signal of these mice is barely observed in the condition of this figure. (**C**) Number of cells/HPF (×400). (**D**) Representative Western blot results of F4/80, iNOS, CX3CR1, CD206 and β-actin are shown. (**E**) Densitometric analysis results were obtained from pooled data. Relative protein expression normalized to β-actin, arbitrary units. (**F**) Immunofluorescence images of phospho-STAT1 (red) and phospho-STAT3 (red) with F4/80 (green) and To-Pro-3 (blue). (**G**) Representative Western blot results of phospho-STAT1, phospho-STAT3 and β-actin are shown. Due to the low expression of F4/80 protein in the skin of B6 and LPS-injected B6 mice, the F4/80 immunofluorescence signal of these mice is barely observed in the condition of this figure. (**H**) Densitometric analysis results were obtained from pooled data. Values represent the mean ± S.E. (n = 4–6). Relative protein expression normalized to β-actin, arbitrary units. *P < 0.05. NS: not significant.

*Flg* ^*ft*^ mouse is a murine model for AD that has IL-17-dependent chronic skin inflammation. The protein expression levels of F4/80, iNOS, CX3CR1 and CD206 increased, and the number of F4/80^+^, iNOS^+^, CX3CR1^+^ and CD206^+^ cells is markedly increased in the skin of *Flg* ^*ft*^ mice (Fig. 5A–E). STAT1 was only weakly phosphorylated, and STAT3 was only modestly phosphorylated in the F4/80^+^ cells of the skin of *Flg* ^*ft*^ mice (Fig. 5F). STAT1 protein was increased and slightly phosphorylated, and STAT3 protein was increased and phosphorylated in the skin tissue of *Flg* ^*ft*^ mice (Fig. 5G,H). The LPS-induced macrophage activation was not deranged in the skin of *Flg* ^*ft*^ mice. These results suggest that macrophages are spontaneously activated in the skin of a murine AD model in a different way from the LPS-induced macrophage activation.

### IL-17A neutralization decreased the protein expression levels of CD206 in the skin of *Flg*^*ft*^ mice

**Figure 6:**
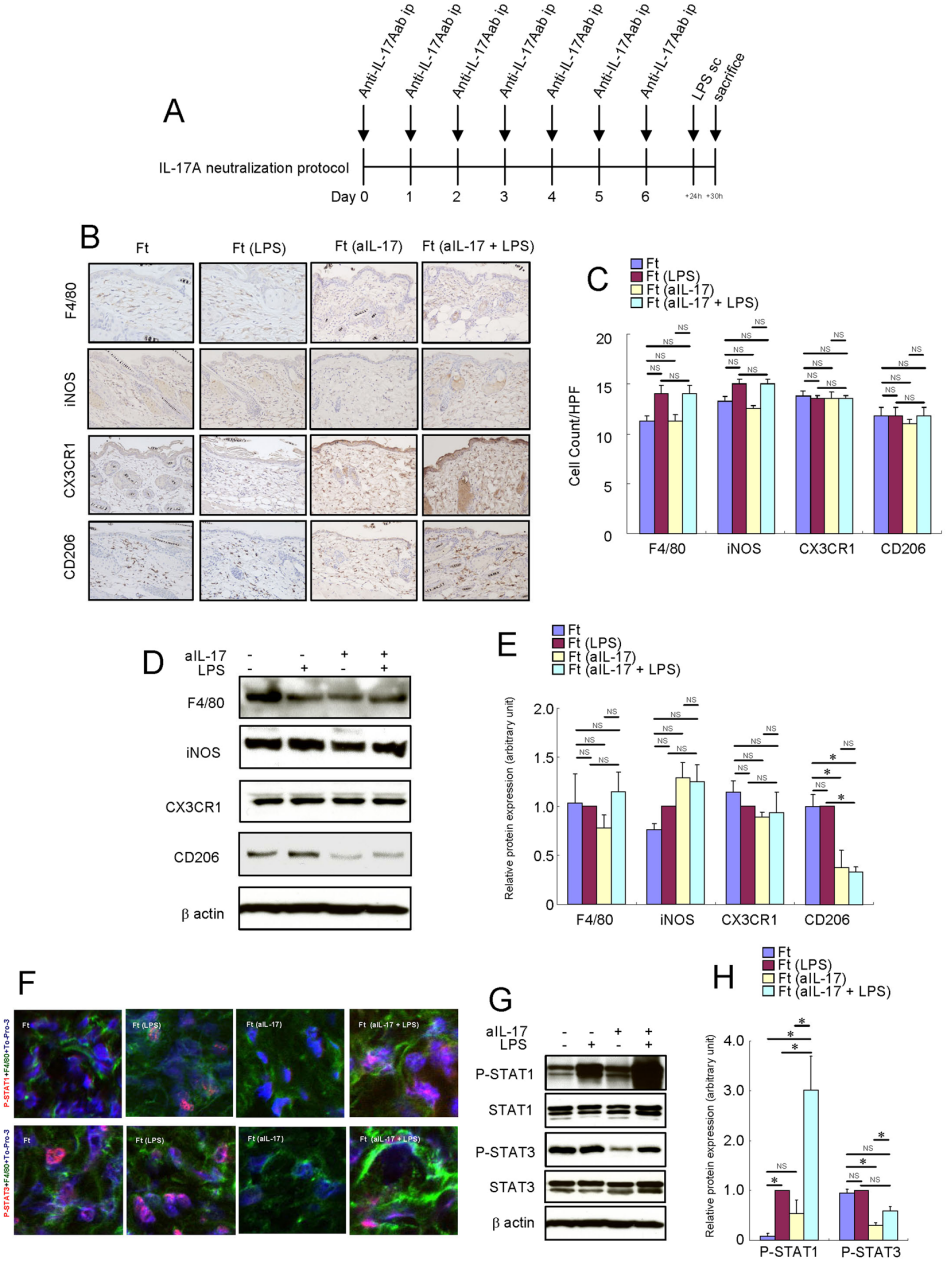
IL-17A neutralization altered the macrophage activation in the skin of *Flg* ^*ft*^ mice. Mouse recombinant anti-mouse IL-17A was administered intraperitoneally (50 μg) once a day for a week in *Flg* ^*ft*^ mice. LPS (0.1 mg) was injected subcutaneously into the dorsal area of the mice. Six hours later, the mice were sacrificed, and the full thickness sections of the dorsal skin were dissected. (**A**) Schema of intraperitoneal IL-17A injection protocol. (**B**) Immunohistochemistry of F4/80, iNOS, CX3CR1 and CD206 protein expression in the skin of *Flg* ^*ft*^ mice. (**C**) Number of cells/HPF (×400). (**D**) Representative Western blot results of F4/80, iNOS, CX3CR1, CD206 and β-actin are shown. (**E**) Densitometric analysis results were obtained from pooled data. Relative protein expression normalized to β-actin, arbitrary units. (**F**) Immunofluorescence co-staining images of phospho-STAT1 (red) and phospho-STAT3 (red) with F4/80 (green) and To-Pro-3 (blue). (**G**) Representative Western blot results of phosho-STAT1, phospho-STAT3 and β-actin are shown. (**H**) Densitometric analysis results were obtained from pooled data. Relative protein expression normalized to β-actin, arbitrary units. Values represent the mean ± S.E. (n = 4–6). *P < 0.05. NS: not significant.

**Figure 7:**
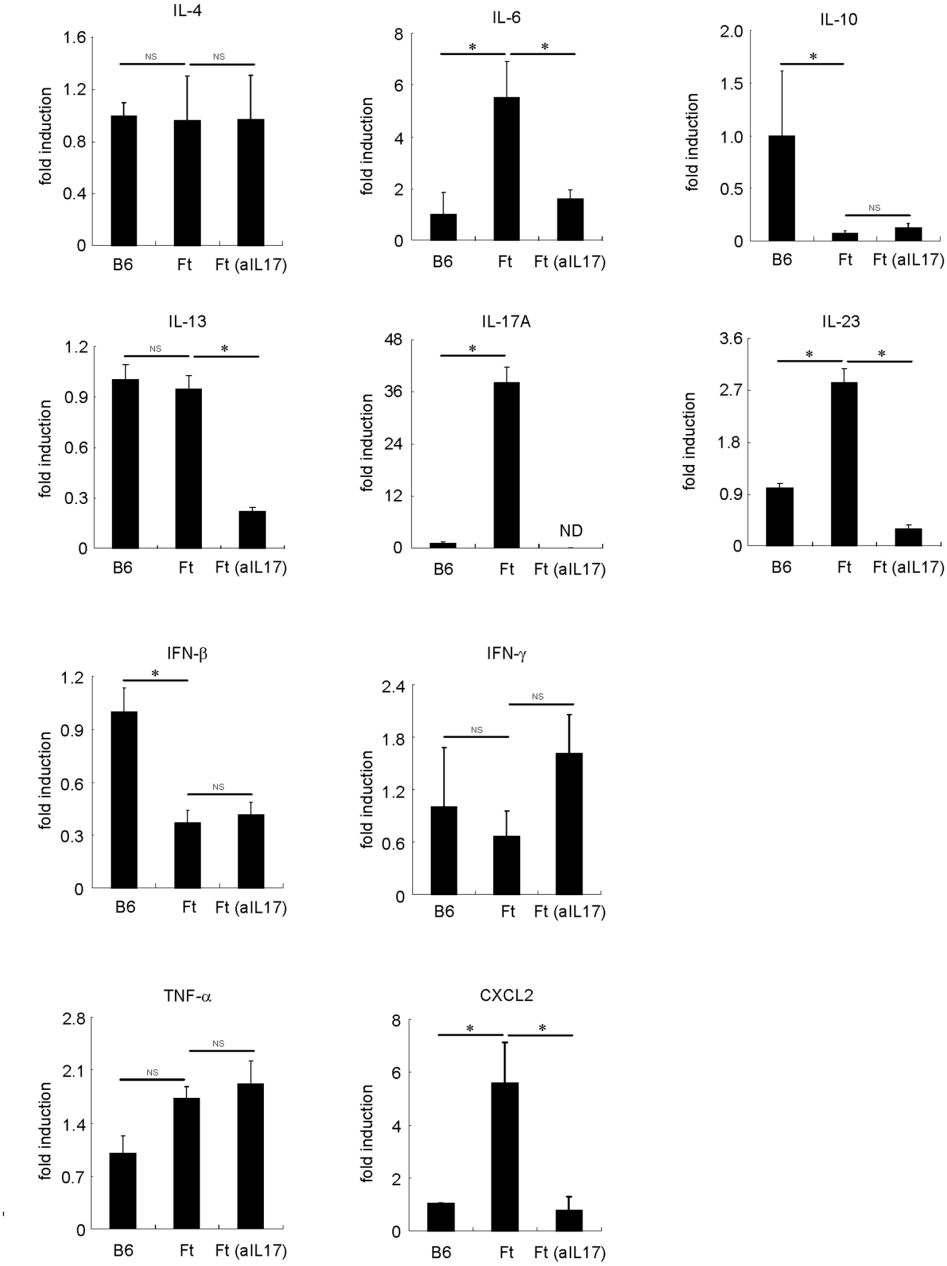
Cytokine, CXCL2 and TNF-α mRNA expression in the skin of *Flg* ^*ft*^ mice and the effects of IL-17A neutralization. Mouse recombinant anti-mouse IL-17A was administered intraperitoneally (50 μg) once a day for a week in the skin of *Flg* ^*ft*^ mice. The mice were sacrificed, and the full thickness sections of the dorsal skin were dissected. Quantitative RT-PCR analysis of cytokine, CXCL2 and TNF-α mRNA expression in the skin of *Flg* ^*ft*^ and B6 mice (n = 4–5). *P < 0.05; *P < 0.005. NS: not significant. ND: no data.

To determine whether the inhibition of IL-17A affects the phenotype of macrophages, we treated the *Flg* ^*ft*^ mice with anti-IL-17A antibody for a week. Treatment with anti-IL-17A antibody did not decrease the number of F4/80^+^, iNOS^+^, CX3CR1^+^ and CD206^+^ cells in the skin of *Flg* ^*ft*^ mice (Fig. 6B,C). Although the protein expression levels of F4/80, iNOS and CX3CR1 were not altered, the protein expression levels of CD206 were suppressed (Fig. 6D,E). Anti-IL-17A antibody treatment did not alter the activation of STAT1, but it reduced the activation of STAT3 in the skin of *Flg* ^*ft*^ mice (Fig. 6F,G,H). Although not statistically significant, anti-IL-17A moderately reduced the activation of STAT3 in the skin of LPS-injected *Flg* ^*ft*^ mice. We examined proinflammatory and proresolving mediators that may alter the macrophage phenotype in the skin of *Flg* ^*ft*^ mice. The expression levels of IL-6, IL-17A, IL-23 and CXCL2 were increased, and the expression levels of IL-10 and IFN-β were decreased in the skin of *Flg* ^*ft*^ mice (Fig. 7). After treatment with anti-IL-17A antibodies, the expression levels of IL-6, IL-13, IL-23 and CXCL2 were decreased in the skin of *Flg* ^*ft*^ mice (Fig. 7). These results suggest that the deranged phenotype of macrophage is mostly independent of IL-17A itself in the skin of *Flg* ^*ft*^ mice.

### iNOS- and CD206-expressing cells in the dermis of psoriasis and AD patients

**Figure 8:**
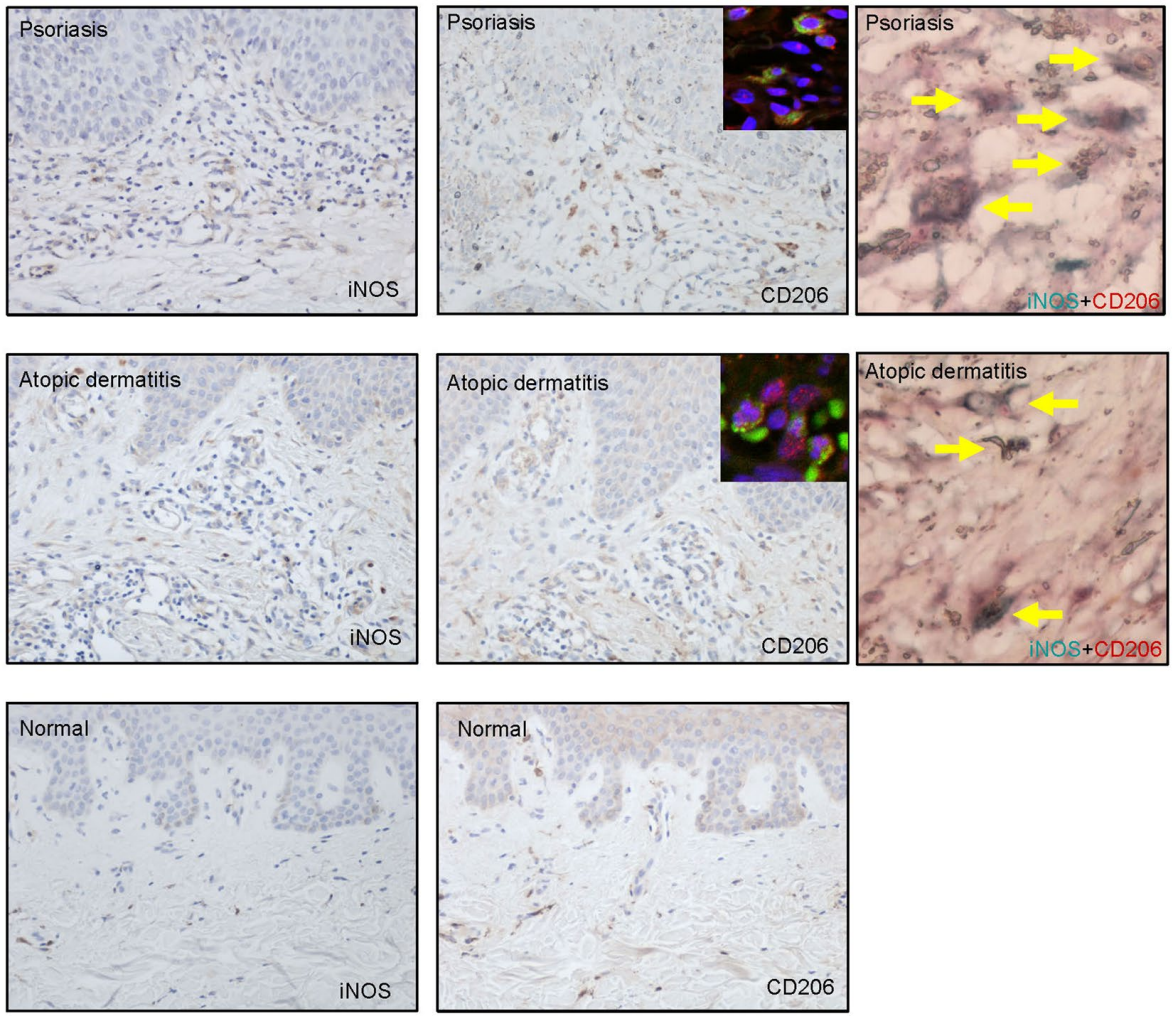
iNOS- and CD206-expressing cells are present in the dermis of psoriasis and AD patients. Representative immunohistochemical analysis (×200) of iNOS and CD206 expression in the skin of psoriasis (n = 3) and AD patients (n = 5), and normal skin (n = 3). Immunofluorescence co-staining images of CD206 (red) with CD68 (green) and colocalization (yellow allow) of CD206 (red) and iNOS (green) protein were shown.

To determine the relevance of the effects of IL-17A in human skin diseases, we evaluated iNOS- and CD206-expressing cells in human psoriasis and AD samples. Immunohistochemical analysis revealed the presence of both iNOS- and CD206-expressing cells in the dermis of psoriasis and AD patients. Some CD206-expressing cells were positive for CD68 and iNOS (Fig. 8).

## Discussion

IL-17 is a key mediator in inflammatory skin diseases. Since macrophages are involved in the IL-17-induced inflammation, we examined the effects of IL-17A on the expression of macrophage activation markers in the skin of mice. Local treatment with subcutaneous injection or systemic treatment with intraperitoneal injection of IL-17A increased the expression levels of F4/80, iNOS, CX3CR1 and CD206 proteins in the skin of B6 mice. IL-17A activated both STAT1 and STAT3 in macrophages in the skin of B6 mice. Likewise, the expression levels of F4/80, iNOS, CX3CR1 and CD206 increased and both STAT1 and STAT3 were activated in the IMQ-treated skin and the skin of *Flg* ^*ft*^ mice. IL-17A neutralization increased iNOS expression and enhanced phosphorylation of STAT1 in the IMQ-treated skin, but it partially decreased CD206 protein expression and prevented phosphorylation of STAT3 in the skin of *Flg* ^*ft*^ mice. No synergistic effects or cross-inhibition of IL-17A and LPS on macrophage activation was observed in the skin of mouse. In the human skin, iNOS^+^ and CD206^+^ macrophages existed in the dermis of psoriasis and AD patients.

Common terms in macrophage phenotyping are ‘classically activated M1 macrophages’ and ‘alternatively activated M2 macrophages’. The F4/80 antigen is a mature mouse cell surface glycoprotein that is highly expressed in macrophages^15^ and can be expressed by both M1 and M2 macrophages. M1 macrophages express iNOS and CX3CR1, and M2 macrophages express CD206^16^. These protein expressions are accompanied by the activation of STAT proteins in macrophages. In general, STAT1 is activated in response to M1-polarizing signals (IL-23, IFN-β and IFN-γ) whereas STAT3 is activated by M2–polarizing signals (IL-4, IL-6, IL-10 and IL-13) in macrophage^17,18^. LPS, the major structural component of the outer wall of Gram-negative bacteria, is a potent activator of macrophages. It was shown that LPS generated free radicals via iNOS and xanthine oxidase in the skin of mice^19^. In this study, we showed that macrophages in the skin of mice were activated by LPS treatment, suggesting that the main source of free radicals is produced from iNOS expressed by LPS-activated macrophages. In general, LPS activates macrophages by TLR4 signaling to direct the macrophage towards the M1 phenotype^16^. However, LPS is known to induce type 2 cytokines, such as IL-6, in macrophages^20^. It is possible that LPS polarizes macrophages directly M1 toward and indirectly M2 toward via IL-6 in the skin of mice. Therefore, it may be reasonable that LPS induced both M1- and M2-related protein expressions in the skin of B6 wild-type mice in our study. A previous report demonstrated synergistic or cross-tolerant effects of IL-17A and LPS on macrophage activation^12,13^. In addition, cross-inhibition of STAT3 between IL-6 and IL10 was reported^21^. Thus, cross talk among different signaling pathways was proposed to affect macrophage phenotype in the skin of mouse. However, our results suggest that IL-17A did not alter the effects of LPS on the heterogeneous macrophage activation in the skin of mouse. These discrepancies may depend on experimental conditions: *in vitro* or *in vivo*, tissue type, dose of LPS, and treatment duration of LPS. Since a maximum dose of LPS was injected according to the previous reports, final conclusion needs further investigations.

Although the phenotypic and functional diversity of the macrophage lineages are becoming widely recognized^22^, there is little known regarding their phenotype in IL-17A-mediated inflammation. A previous report demonstrated the direct activation of macrophage by IL-17A^23^, but macrophage differentiation is mainly regulated by T cell-derived cytokines such as IFN-γ, IL-4, IL-6, IL-10 and IL-13. In our study, both M1 and M2 macrophage-related proteins were induced by IL-17A. *In vivo* IL-17A-induced inflammation may directly and indirectly involve the activations of these STAT proteins in macrophages. Previous reports demonstrated that IL-17A activated both STAT1 and STAT3 in human monocytic leukemia cells and keratinocytes and human keratinocytes^24,25^, implying that IL-17A can induce M1 and M2 macrophage-related proteins via direct activation of both STAT1 and STAT3 in the skin of mouse. On the other hand, IL-17A is known to act on a variety of *in vivo* cells and enhance both M1 macrophage-polarizing signal (IFN-γ) and M2 macrophage –polarizing signal (IL-6)^26^. These inflammatory cytokines could concomitantly activate both STAT1 and STAT3 in the macrophages. Previous studies outside the dermatological fields have demonstrated the controversial effects of IL-17 on macrophages. IL-17 expression was shown to increase the M1/M2 macrophage ratio at the local mucosal site of both murine and human bisphosphonate-related osteonecrosis of the jaw lesion^27^. IL-17 induced an atypical M2-like macrophage to regulate intestinal inflammation^28^ but it induced an M1-like phenotype in *in vitro* human macrophages^12^. IL-17A monoclonal antibody treatment prevented the progression of advanced atherosclerotic lesions and induced plaque stability in mice by interfering with the activity of macrophages^12^. The direct treatment of *in vitro* mouse macrophages with IL-17A induced unique cytokine profiles that included GM-CSF, IL-3, IL-9, CCL4/MIP1β, CCL5/RANTES and IL-12p70^23^. The induction of IL12p70 favors the induction of Th1 differentiation in response to transgenic CD4^+^ T cells. These macrophage-derived cytokines may interfere with the M1/M2 macrophages ratio by an autocrine signaling pathway. Thus, the phenotype of macrophages under the influence of IL-17A may depend on the environment of the organ or tissue. We have shown for the first time that IL-17A induced M1/M2 heterogenous macrophages in the skin of mouse.

IMQ-induced skin inflammation mimics the symptoms of psoriasis such as skin thickening, scaling, erythema and microabscesses^29^. Topical application of IMQ activates TLR 7/8 of Th17 cells, plasmacytoid dendritic cells and γδT cells to produce IL-17A^30,31^. We demonstrated that macrophages were activated in the IMQ-treated skin of mice. The direct activation of macrophages via TLR 7/8 by IMQ, a primary innate immune mechanism, occurs within 3–5 days after IMQ application. However, 7 days of consecutive application may elicit the cycle of antigen transport to drain the lymph nodes, promote antigen-presenting T-cell interactions, induce clonal expansion, and stimulate Th17 cells migration towards the damaged skin tissue. Thus, our data suggest that adaptive Th17 immunity contributes to the activation of macrophages in IMQ-induced skin inflammation. In our study, the protein expression levels of F4/80, iNOS, CX3CR1 and CD206 were elevated in the IMQ-induced skin of mice. Furthermore, both STAT1 and STAT3 were activated in the skin of these mice. These results suggest that macrophages with a heterogeneous M1/M2 phenotype were activated in the IMQ-treated skin of adaptive Th17-induced inflammation. Morimura *et al*. also recently reported that CX3CR1^+^ macrophages were partially involved in the IMQ-induced skin inflammation^32^, suggesting the importance of macrophage activity in this psoriasis mouse model.

We used *Flg* ^*ft*^ mice as a model for IL-17A-induced chronic skin inflammation. *Flg* ^*ft*^ mice arose spontaneously in 1958 at the Jackson Laboratory and were first reported in 1972^33^. A filaggrin deficiency in their epidermis was discovered in 2000 that were due to mutations in the filaggrin and matted genes^9,34,35^. Filaggrin deficiency causes the disruption of skin barrier functions^36^ and induces AD-like immune responses in the skin of *Flg* ^*ft*^ mice^37^. Human AD is classically characterized by a Th2-dominant condition. However, it has been reported that levels of IL-17A, but not IL-4 and IL-13, is elevated in the skin of *Flg* ^*ft*^ mice^38^. Thus, *Flg* ^*ft*^ mice have been used to investigate the effects of IL-17A in skin inflammation^10^. In our study, in addition to the elevated protein expression levels of F4/80, iNOS, CX3CR1 and CD206, both STAT1 and STAT3 were activated in the skin of *Flg* ^*ft*^ mice. These results suggest that the activated macrophages had a M1/M2 heterogeneous phenotype in the skin of AD.

Real-time RT-PCR analysis indicated that the expression levels of IL-6, IL-17A, IL-23 and CXCL2 were elevated in the IMQ-treated skin and the skin of *Flg* ^*ft*^ mice. IL-23 promotes the development of an IL-17-producing CD4^+^ T cell subset^39^. Since IL-17A appeared to be a downstream factor of IL-23, we examined the effects of IL-17A on macrophages in the skin of B6 wild-type mice. Both intraperitoneal and subcutaneous injection of IL-17A induced the protein expression of F4/80, iNOS, CX3CR1 and CD206. Furthermore, STAT1 was slightly activated and STAT3 was markedly activated in the IL-17A-treated skin of B6 mice, mimicking the IMQ-treated skin and the skin of *Flg* ^*ft*^ mice. IL-17A neutralization suppressed the expression of IL-17A, IL-23 and CXCL2 in the IMQ-treated skin. On the contrary, in addition to suppressing the expression of IL-6, IL-13, IL-23 and CXCL2 in the skin of *Flg* ^*ft*^ mice, IL-17A neutralization reduced the LPS-induced IL-4 expression, normalized IL-23 expression and enhanced IFN-γ expression (data not shown). Along with the increase in iNOS protein expression and STAT1 phosphorylation in the skin of IMQ-treated mice or the decrease in CD206 protein expression and STAT3 dephosphorylation in the skin of *Flg* ^*ft*^ mice, IL-17A neutralization may shift macrophages from the M2 to the M1 phenotype in the skin of mouse. Moreover, these results suggest that IL-6 and IL-13 were important for activating STAT3 and M2-polarizing of the macrophages in the skin of mouse. The discrepant effects of IL-17A neutralization between IMQ-treated mice and *Flg* ^*ft*^ mice may be due to differences in the pathophysioloy of these models.

In human IL-17A-related skin diseases, such as psoriasis and AD, CD206 is a specific marker for mature macrophages in the skin. CD68^+^ and CD163^+^ macrophages express CD206 in the skin of AD^40^ and psoriasis patients^41^, respectively. We have demonstrated for the first time the presence of iNOS^+^ and CD206^+^ macrophages in the skin of patients with psoriasis and AD. However, correlations between mouse and human macrophages and their representative subtypes are poor and are major barriers to understanding human immunity^42^. Therefore, these results need to be further investigated in models that are more representative of the human physiology.

Our findings demonstrated the interactions between IL-17A signaling and inflammation-dependent regulation of macrophage function in the skin *in vivo*. We have provided evidence that there is a heterogeneous activation of macrophages in IL-17A-induced skin inflammation.
